# Post-vaccination outcomes in association with four COVID-19 vaccines in the Kingdom of Bahrain

**DOI:** 10.1038/s41598-022-12543-4

**Published:** 2022-06-02

**Authors:** Manaf AlQahtani, Xing Du, Sujoy Bhattacharyya, Abdulla Alawadi, Hamad Al Mahmeed, Jaleela Al Sayed, Jessica Justman, Wafaa M. El-Sadr, Jack Hidary, Siddhartha Mukherjee

**Affiliations:** 1 Bahrain Defense Force Hospital, National Task Force for Combating COVID-19, Riffa, Bahrain; 2 Division of Oncology, Department of Medicine, Columbia University, New York, USA; 3 School of International and Public Affairs, Columbia University, New York, USA; 4 Mailman School of Public Health, ICAP at Columbia University, New York, USA; 5 Sandbox@Alphabet, Mountain View, CA USA

**Keywords:** Diseases, Health care, Medical research

## Abstract

With the emergence of new SARS-Cov2 variants, critical questions have arisen about: (1) the effectiveness of the available COVID-19 vaccines developed to protect against the original Wuhan (wild type) variant and (2) the magnitude and clinical consequences of post-vaccination infections in the context of the Delta variant of SARS-Cov2. While some “real world” experiences with various vaccines have been reported, to our knowledge, few have examined comparative outcomes of various vaccines in one country as new SARS-CoV-2 variants have emerged. Here we present an analysis of COVID-19 related outcomes from a national database in Bahrain, a country with a total population of 1.51 million, where four vaccines were deployed (total vaccinated = 1,003,960 adults): AstraZeneca (AZ/Covishield), Pfizer/BioNtech, Sinopharm and Sputnik V. We compare the four vaccines, based on the following post-vaccination outcomes: SARS-CoV-2 infections, hospitalisations, ICU admissions and deaths, compared to unvaccinated individuals. We conclude that the four vaccines used in Bahrain were effective in significantly reducing all four COVID-19 related outcomes compared to unvaccinated individuals, prior to, and during the period when the Delta variant predominated in the country. However, compared to the three other vaccines, individuals vaccinated with Sinopharm vaccine had a higher risk of post-vaccination infections, hospitalisations and ICU admissions (e.g., 6.94%, 2.24%, 1.99% and 1.52% of COVID-19 cases of Sinopharm, Sputnik V, Pfizer and Covishield recipients, respectively, required hospitalisation versus 13.66% of COVID-19 cases among unvaccinated individuals); however, given the confounding factors, this needs to be confirmed by further studies. We find no evidence of biased selection for any vaccine, but note waning protection of the Pfizer/BioNtech vaccine during the January to June 2021 period in the age > 60 y cohort; however, this cannot be distinguished from the overall fall in hospitalisations overall. Our findings support the value of vaccination in preventing COVID-19 related outcomes, provide real world estimates on the outcomes and frequencies of post-vaccination infections for the four vaccines, which may inform vaccine selection in the context of the Delta variant across the globe.

## Introduction

Vaccination offers a critical tool for the control of the COVID-19 pandemic. To date, multiple vaccines have been developed that utilize different technologies^1–5^. Clinical trials have demonstrated variable levels of protective efficacy against symptomatic COVID-19, the primary outcome for many of these studies. However, these clinical trials were conducted at different times during the pandemic, in different countries and with varying prevailing SARS-CoV-2 variants. There have been no direct head-to-head comparisons of efficacy across vaccines and only limited comparisons of effectiveness in a real-world context as new variants of concern have emerged^6^.

Breakthrough infections after vaccination or natural infection have been reported for several viruses and bacteria^7,8^. With regards to COVID-19, several studies have described post vaccination infections, although symptoms and hospitalisations were less frequently reported among the vaccinated compared to the unvaccinated. For example, in two states in the U.S., between July and September 2021, COVID-19-related deaths were seven times less frequent among the vaccinated compared to the unvaccinated^9,10^. More recently, the absolute risk of testing positive for SARS-CoV-2 after vaccination was estimated as approximately 1% among health care workers at two sites in California during an eight-week period after receipt of one or two doses of either the Pfizer or Moderna vaccines^11^. Finally, two studies from Israel documented breakthrough infections post-vaccination. The first study reported 39 infections in 1497 HCWs fully vaccinated with Pfizer/BioNtech, with 85% of the cases due to the Alpha variant^12^. The second study that vaccine recipients who tested positive at least 7 days after the second dose of the vaccine were largely infected with B.1.351, while individuals tested between 2 weeks after the first dose and 6 days after the second dose were infected by B.1.1.7^13^. However, both studies were performed with a single vaccine at relatively few sites, and both recruited a majority of patients before the emergence and dominance of the Delta variant.

Comparative post-vaccination outcome data have been limited with few reports available from Chile^14^ (from February to early July 2021) and from Qatar^15^ (from December to July 2021), describing the effectiveness of vaccines administered in each country (Sinovac, Pfizer-BioNTech and AstraZeneca; and Pfizer-BioNTech and Moderna, respectively), with the Pfizer-BioNTech vaccine proving to be the most effective at preventing symptomatic COVID-19 in Chile, and Moderna vaccine showing higher effectiveness against severe, critical or fatal COVID-19 disease due to Delta variant in Qatar. At the same time, a recent study from Israel has raised the prospect of waning of protection from the Pfizer/BioNtech vaccine over time, with reported increase in post vaccination infections observed in conjunction with the increased prevalence of the Delta variant in the country^16^. Notably, post-vaccination infections and hospitalisation with the Delta variant were associated with the length of time that had elapsed since receipt of vaccination. However, the latter findings have not been confirmed in other studies^17^. Thus far, data with regards to durability of protection are needed in relation to some of the other commonly deployed across the globe, e.g., AZ/Covishield and Sinopharm. These questions have reached great urgency as countries make decisions about how various vaccines are likely to perform, particularly in the context of the rise of the Delta variant and whether additional (i.e., booster) doses of vaccines are necessary.

The Government of Bahrain’s decision to deploy four vaccines provided the opportunity to examine and compare the relative effectiveness of several vaccines in terms of COVID-19 cases, hospitalisations, ICU admissions and deaths over time in a large population as new variants were emerging. The findings from this study will inform countries as they make decisions regarding vaccination procurement and implementation.

## Results

### Vaccination uptake

**Figure 1:**
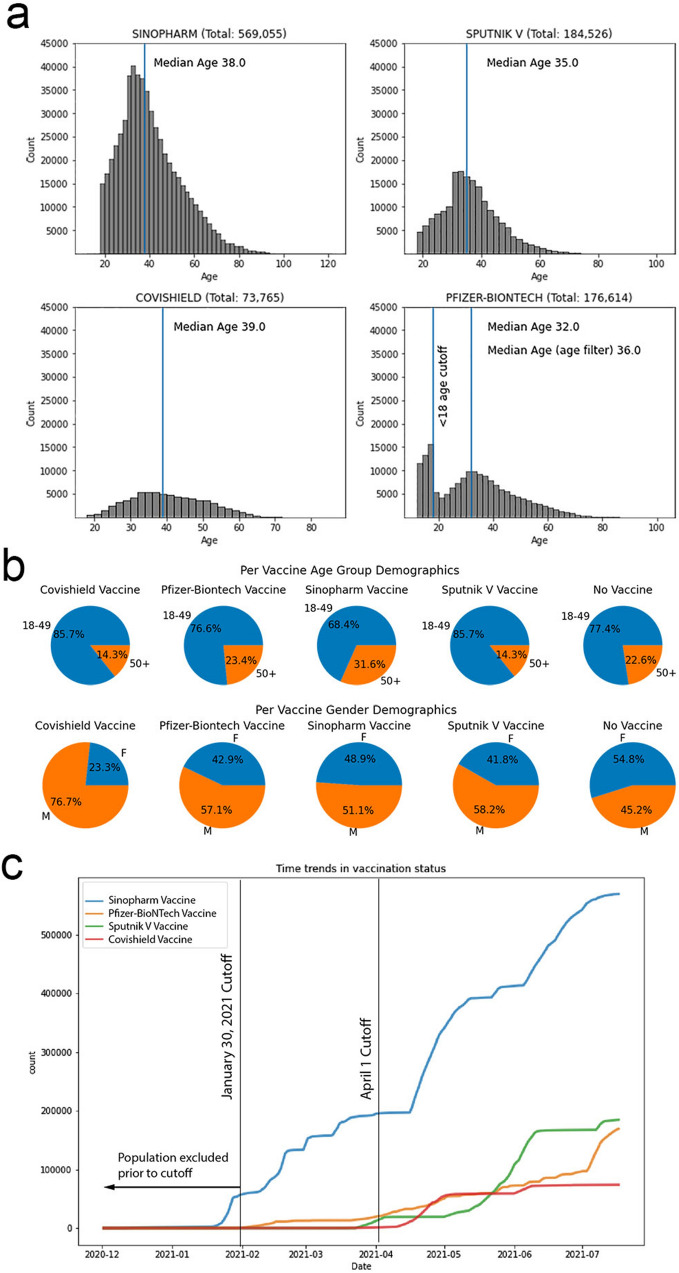
Distribution of Covid vaccines. (**a**) Age distributions of the four different vaccines administered in the population. Pfizer-BioNTech was the only vaccine given to ages 12–18 < 18 years of age, and those data were excluded from the analysis. (**b**) Age and gender demographics for all vaccines. Note that all individuals less than 18 years were eliminated from these pie charts and from the analysis below. (**c**) Visualization of the vaccine rollout. The curves display the counts of individuals that have presumptive protection (i.e. 14 days after the second booster dose) for each of the four vaccines on each day. January 30th is the date that the first Pfizer recipients achieved presumptive protection (i.e. 14 days after their second Pfizer vaccination dose) and is therefore used as a cutoff to censor any early recipients of Sinopharm. April 1 is the cutoff date for the deployment of the AZ/Covishield and Sputnik V vaccine, and was similarly used in any comparison between these vaccines and Sinopharm (see Supplementary Table 2).

Between December 9, 2020 and July 17, 2021, a total of 569,054 individuals were vaccinated with Sinopharm, 184,526 with Sputnik, 73,765 with AstraZeneca (AZ/Covishield) and 169,058 with Pfizer/BioNtech vaccines, while 245,876 remained unvaccinated (Fig. 1). The median and mean age of vaccinated versus unvaccinated individuals are also shown in Fig. 1a. The unvaccinated cohort included travelers, asymptomatic and symptomatic contacts of those who had tested positive for SARS-COV2, individuals who had symptoms consistent with SARS-CoV2 infection, those undergoing operative and elective medical procedures, and individuals with positive tests identified through random testing in areas of high population density. We excluded individuals between 12–17 years of age who received the Pfizer/BioNtech vaccine. Approximately 250,000 children < 12 years of age were also excluded from all the cohorts as they were not eligible for vaccination. Thus, the total number in the analytic cohort, including vaccinated and unvaccinated individuals, was 1,242,279. The total population of the nation is 1.51 million, and accounting for the 250,000 children excluded from analysis, we note that 17,721 (1.17% of total) individuals had no records available in the database. We also note that approximately 2000 individuals above age 80 received the Sinopharm vaccine, while the numbers above age 80 receiving the other vaccines were in the 100 s. We acknowledge that this could represent a potential cofounder. However, detailed analysis of the breakthrough vaccinations causing deaths did not reveal that this bias was relevant, since the deaths were distributed across ages.

We determined the date of achievement of presumptive protection (PP), defined as the date corresponding to 14 days after receipt of the second dose of any of the four vaccines, as each involved a two-dose regimen. As noted in Fig. 1c, the Sinopharm vaccine was the first and most frequently used vaccine in Bahrain (total number of recipients: 569,054; date of PP of first vaccine recipients: December 9, 2020; date of 50% rollout: April 24, 2021). Pfizer/BioNtech vaccine was utilized next (total number of recipients 169,058; date of PP of first vaccine recipients: Jan 30, 2021, date of 50% rollout: June 14, 2021) followed by use AZ/Covishield vaccine (total number of recipients: 73,765; date of PP of first vaccine recipients: March 13, 2021, date of 50% rollout: April 24, 2021), and lastly by Sputnik V (total number of recipients: 184,526; date of PP of first vaccine recipients: March 4, 2021, date of 50% rollout: May 30, 2021).

### Characteristics of vaccine recipients

The median age of the population in Bahrain is 32.5 years, and, initially, people over 50 were given priority in vaccination. The median age of individuals vaccinated was similar across the four vaccines utilized, Sinopharm: 38 years, AZ/Covishield: 39 years, Sputnik V: 35 years, Pfizer/Biontech: 36 years (vaccinated individuals 12–17 years were censored). There were, however, differences in the sex distribution. While with Pfizer/BioNtech and Sinopharm vaccines, there was a similar male to female distribution (63.4% males/36.6% females and 60.9% males/39.1% females respectively), men were more likely to have received the Sputnik V and AZ/Covishield vaccines with 73.4% male/26.6% female and 81.9% males/18.1% females, respectively (Fig. 1b). In the unvaccinated cohort, individuals between the ages of 0–17 were overrepresented since vaccination was not recommended for such individuals with the exception of the Pfizer/BioNtech vaccine. However, all those < 18 years of age were excluded from the analysis and from Fig. 1. The last panel of Fig. 1b shows the distribution of unvaccinated individuals, excluding those for whom vaccines were unavailable (ages 0–17), or were restricted for those 16 and 17 years old for Pfizer/BioNtech vaccination.

### SARS-Cov2 PCR testing strategy and coverage

Beginning in February 2020, the COVID-19 Task Force of Bahrain began testing all arriving travelers into the country, suspected cases, symptomatic individuals, asymptomatic contacts (including family members) of those who had tested positive for SARS-CoV-2, all hospitalized and critically ill patients suspected of being infected with SARS-Cov2, and through large-scale random testing of individuals (described below and in “Methods”). SARS-CoV-2 testing expanded substantially after December 1, 2020 and reached a peak average of 15,000 tests per day by February 2021. In April 2021, SARS-CoV2 testing was further expanded with the addition of a targeted strategy—namely, focusing on men and women who recently arrived from high burden countries, as well as symptomatic individuals and populations considered at high-risk (i.e., residents in high density areas, front-line and health-care workers (HCW) and elderly persons), with particular attention to those who required hospitalisation, ICU admission or died.

Since February 2021, a total of 2.6 million tests have been performed among approximately 80% of the population, (i.e., 3.6 tests per person), with half consisting of tests performed on randomly selected individuals and at an overall positivity of 5%.

During April, May and June 2021, Bahrain conducted between 12,000 and 15,000 PCR tests daily (i.e., 0.8–1.0% of the total population were tested per day). In addition, since March, rapid antigen tests, which are not included in the official testing count, became widely available in pharmacies across the country at subsidized prices and persons who tested positive with a rapid test reported their results on a mobile application and were required to undergo confirmatory PCR testing.

### Genetic sequencing of SARS-CoV-2 isolates

**Figure 2:**
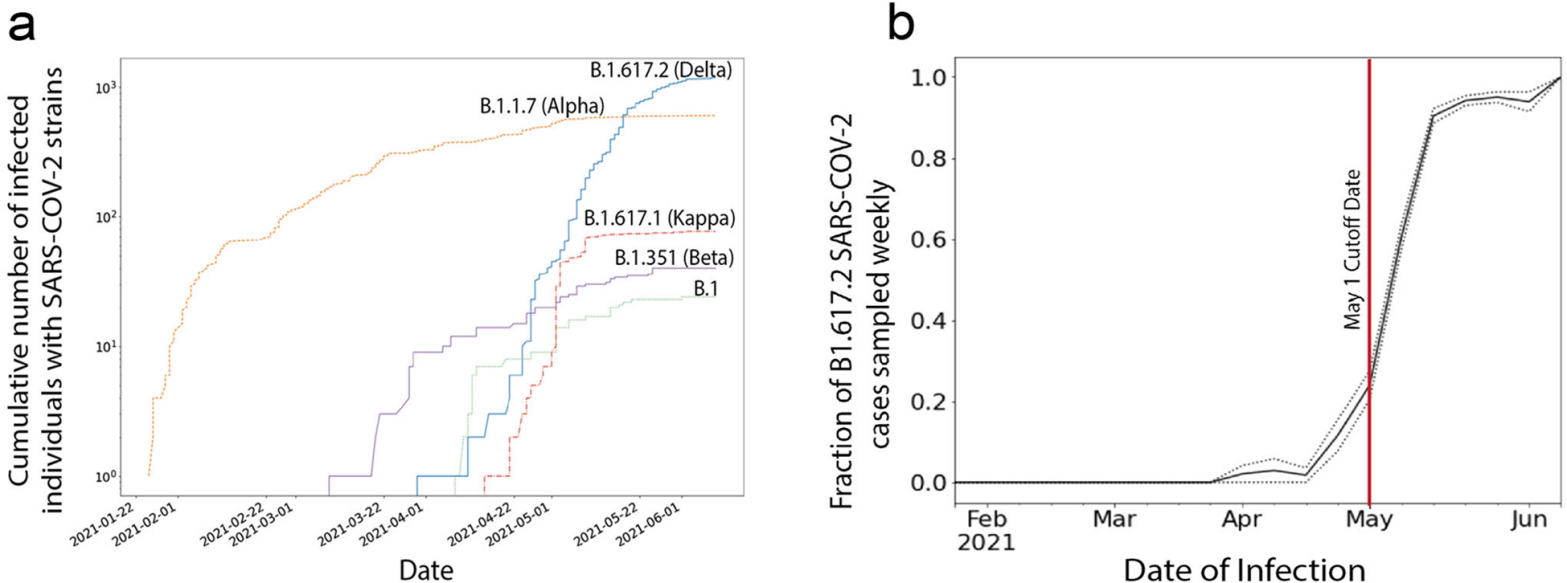
Various strains of SARS-Cov2 in Bahrain. (**a**) Cumulative counts of individuals infected with various strains of SARS-Cov2 in Bahrain. (**b**) Fraction of infections of Delta/B.1.617.2 (over all other variants detected) with +—one s.e. of the total fraction. Total counts of B.1.617.2 exceed other detections (note log scale). May 1 (red line) was selected as a cutoff date of when B.1.617.2 began to rise as the dominant strain in the population.

Beginning in June 2020, genetic sequencing was initiated on SARS-CoV-2 isolates from selected travelers, symptomatic cases and critically ill patients and post vaccination cases. Sequencing expanded in February 2021 and has continued; we provide data until July 29, 2021, by which time a total of 7807 viral isolates had been sequenced. Isolates were initially dominated by the Beta/B.1.351 variant between March and April 2021 (Fig. 2a), followed by a rise in Delta/B.1.617.2 and Kappa/ B.1.617.1 variants between April through June 2021. The prevalence of the Delta/B.1.617.2 variant continued to rise from April through July 2021. By May 1, 2021, twenty-five percent of the isolates were Delta variant (Fig. 2b) and, thus, in subsequent analyses, we use May 1 (red line, Fig. 2b) as the starting point for the rise of the Delta variant.

### COVID-19 outcomes

There was a total of 180,840 individuals who had a PCR positive test result noted in the cohort, with 134,728 among those 18 years and older. There were 13,105 hospitalisations, including 1,636 ICU admissions, and a total of 1,030 deaths. Using pairwise comparisons, we found significantly higher percent hospitalisation, ICU admissions and deaths in the unvaccinated group compared to the vaccinated group for each of the four vaccines (Tables 1, 2, 3, 4, 5, 6). For example, the percent of COVID-19 cases who died among unvaccinated individuals > 50 years of age was 3.83 and 7.49 fold higher compared to recipients of Sinopharm and Sputnik V, respectively. There were no deaths in the AZ/Covishield vaccinated group. In individuals ≤ 50 years, the percent of COVID-19 cases who died among unvaccinated people was 8.07 fold higher compared to Sinopharm vaccine recipients. There were no deaths in this age group among Sputnik V and Pfizer/BioNtech vaccine recipients, and 1 death among the AZ/Covishield vaccine recipients (Table 4).

**Table 1:** Infection rates by vaccine (events per 100 k per week).

	Total	OR	Above 50	OR	Below 50	OR
Unvaccinated	780.50	N/A	980.08	N/A	765.11	N/A
Sinopharm	462.96	1.66	490.17	1.93	452.94	1.66
Pfizer	194.19	3.68	188.53	5.02	195.77	3.53
Covishield	336.12	2.12	321.67	3.09	340.06	2.00
Sputnik	317.87	2.41	328.80	3.02	316.78	2.36

*In pairwise comparison to unvaccinated, all p values < 0.001.

**Table 2:** Hospitalisation (%) of SARS-COV-2 PCR positive cases (18 + Ages) after Jan 30th.

Vaccine type	Total rate (hospitalisation)			> 50 rate (hospitalisation)			< 50 rate (hospitalisation)		
	Rate (Total)	LCL UCL	OR	Rate (Total)	LCL UCL	OR	Rate (Total)	LCL UCL	OR
Unvaccinated	13.66 (8340)	13.30 14.02	NA	32.79 (3440)	31.62 33.98	NA	9.69 (4900)	9.35 10.03	NA
Sinopharm	6.94 (1683)	6.53 7.37	2.12	16.1 (1081)	14.97 17.27	2.54	3.43 (602)	3.09 3.8	3.02
Sputnik V	2.24 (77)	1.66 2.96	6.89	11.87 (45)	8.07 16.62	3.62	1.05 (32)	0.65 1.6	10.13
Pfizer-BioNtech	1.99 (40)	1.30 2.91	7.78	4.85 (21)	2.67 8.01	9.57	1.21 (19)	0.64 2.07	8.78
Covishield	1.52 (45)	1.02 2.18	10.2	4.19 (24)	2.4 6.73	11.16	0.88 (21)	0.48 1.48	12.05

*In pairwise comparison to unvaccinated, all p values < 0.001.

**Table 3:** ICU admission (%) of all SARS-Cov2 positive cases (18 + Ages) after Jan 30th.

Vaccine type	Total rate (ICU)			> 50 rate (ICU)			< 50 rate (ICU)		
	Rate (total)	LCL UCL	OR	Rate (total)	LCL UCL	OR	Rate (total)	LCL UCL	OR
Unvaccinated	2.02 (1232)	1.87 2.17	NA	7.55 (792)	6.90 8.23	NA	0.87 (440)	0.77 (0.98)	NA
Sinopharm	0.57 (138)	0.45 1.70	3.6	1.7 (114)	1.32 2.14	4.72	0.14 (24)	0.08 0.22	6.4
Sputnik V	0 (0)	0.00 0.11	inf	0 (0)	0.00 1.03	inf	0 (0)	0.00 0.13	inf
Pfizer-BioNtech	0.05 (1)	0.00 0.32	41.3	0.23 (1)	0.01 1.47	35.3	0 (0)	0.00 0.25	inf
Covishield	0.1 (3)	0.02 0.34	20.3	0.17 (1)	0.01 1.11	46.7	0.08 (2)	0.01 0.35	10.4

*In pairwise comparison to unvaccinated individuals all p-values are < 0.001. Inf refers to an infinite OR, since the rate was 0.

**Table 4:** Deaths (%) of all SARS-Cov2 positive cases (18 + Ages) after Jan 30th.

Vaccine type	Total rate (death)			> 50 rate (death)			< 50 rate (death)		
	Rate (total)	LCL UCL	OR	Rate (total)	LCL UCL	OR	Rate (total)	LCL UCL	OR
Unvaccinated	1.33 (810)	1.21 1.45	NA	5.65 (593)	5.09 6.25	NA	0.43 (217)	0.36 (0.51)	NA
Sinopharm	0.46 (112)	0.36 0.58	2.9	1.53 (103)	1.18 1.95	3.8	0.05 (9)	0.02 0.11	8.4
Sputnik V	0.09 (3)	0.01 0.29	15.4	0.79 (3)	0.13 2.65	7.5	0 (0)	0.00 0.13	inf
Pfizer-BioNtech	0.15 (3)	0.02 0.50	9.0	0.69 (3)	0.11 2.32	8.6	0 (0)	0.00 0.25	*inf
Covishield	0.03 (1)	0.00 0.22	39.7	0 (0)	0.00 0.68	inf	0.04 (1)	0.00 0.27	**10.3

Death rates% as percentages of PCR positive cases in populations broken down by vaccine type, and age > 50 and < 50 over the time period of Jan 30th 2020—July 17th 2021. Tables for April 1-July 17th (when the AZ/Covishield and Sputnik V vaccines were deployed) are in Supplementary figures. Only vaccinated cases that tested positive 14 days after their final dose were included. UCL refers to Upper Confidence Limit while LCL refers to Lower Confidence Limit.

*P-value is 0.029. ** P-value is 0.0019. All other p-values are < 0.001 in pairwise comparisons to unvaccinated individuals.

**Table 5:** Comorbidities present in vaccinated individuals who died from post-vaccination infections*.*

	Total Deaths	Fully vaccinated before death	Obesity (percent of total deaths per vaccine)	HTN (percent of total deaths per vaccines)	DM (percent of total deaths per vaccines)	CV disease (percent of total deaths per vaccines)	Renal disease (percent of total deaths per vaccine)	Lung disease (percent of total deaths per vaccine)
Sinopharm	196	125	26 (13%)	120 (61%)	108 (55%)	10 (5%)	30 (15%)	14 (7%)
Pfizer-BioNtech	9	3	0	7 (78%)	7 (78%)	0	4 (44%)	0
Covishield	4	1	1 (25%)	2 (50%)	2 (50%)	0	0	0
Sputnik	21	2	2 (9.52%)	6 (28%)	5 (24%)	0	0	2 (9.5%)

Not all comorbidities were recorded, but the major comorbidities are represented in the chart. *HTN* hypertension, *DM* diabetes mellitus, *CV* cardiovascular disease (except HTN).

**Table 6:** Pairwise comparison (events per 100,000 persons per week) of the Pfizer/BioNtech and the Sinopharm vaccines (a) and Unvaccinated versus Sinopharm vaccinated individuals (b) in age cohorts > 50 and < 50 showing the rates of infections, hospitalisations, ICU admissions and deaths, p values and odds ratios.

	Pfizer-BioNTech vs Sinopharm (after Jan 30)											
	ALL				Over 50				Under 50			
	Rate (Pfizer)	Rate (Sino)	p	OR	Rate (Pfizer)	Rate (Sino)	p	OR	Rate (Pfizer)	Rate (Sino)	p	OR
Infections	136.28	350.53	< .001	0.44	128.44	366.57	< .001	0.39	139.65	345.52	< .001	0.46
Hospitalisations	2.82	28.36	< .001	0.13	6.59	62.11	< .001	0.12	1.74	14.74	< .001	0.17
ICU admissions	0.08	2.29	< .001	0.04	0.36	6.62	< .001	0.06	0.0	0.49	0.034	0.00
Deaths	0.22	1.64	< .001	0.15	0.96	5.45	< .001	0.18	0.0	0.16	**NS** **0.16**	0.00

	Unvaccinated vs Sinopharm (after Jan 30th)											
	ALL				Over 50				Under 50			
	Rate (Unvax)	Rate (Sino)	p	OR	Rate (Unvax)	Rate (Sino)	p	OR	Rate (Unvax)	Rate (Sino)	p	OR
Infections	642.96	350.53	< .001	1.72	781.32	366.57	< .001	2.02	631.66	345.52	< .001	1.70
Hospitalisations	51.06	28.36	< .001	1.78	225.55	62.11	< .001	3.49	33.98	14.74	< .001	2.35
ICU admissions	6.39	2.29	< .001	2.70	41.44	6.62	< .001	5.92	2.65	0.49	** < .001**	5.09
Deaths	4.42	1.64	< .001	2.72	37.17	5.45	< .001	6.56	1.35	0.16	**NS** **0.07**	7.65

All rates are events per 100,000 of the sample population. Note that the p values for the comparison were all < 0.01, except ICU admissions in “under 50” cohort and deaths for the Sinopharm versus Pfizer comparison and Sinopharm versus Unvaccinated comparison (in bold). Note that early recipients of Sinopharm (Dec 9th–Jan 30th) were eliminated from the analysis, since Pfizer vaccination began on Jan 30th. Comparisons between vaccines between April 1–July 17th (when AZ/Covishield and Sputnik V were deployed) are in Supplementary Tables.

Despite the overall effectiveness of all four vaccines in decreasing the risk of COVID-19 related hospitalisations, intensive care unit admissions and deaths, we noted a significantly higher risk of SARS-CoV-2 infection, hospitalisations and ICU admissions among recipients of the Sinopharm vaccine compared to other vaccine recipients. For example, the percentage of deaths among all post-vaccination COVID-19 cases among recipients of the Sinopharm vaccine was 0.46% (i.e., 112 deaths) versus 0.15% for Pfizer/BioNtech (i.e., 3 deaths) and 0.03% for AZ/Covishield (1 death) (Tables 1, 2, 3, 4). This trend was consistent for all COVID-19 outcomes, i.e. infection, hospitalisation, ICU admission and death (Tables 1, 2, 3, 4).

To further examine this issue in view of early use of Sinopharm vaccine indicated above prior to the introduction of other vaccines, we focused on comparing outcomes with recipients of Pfizer/BioNtech vaccine during the period of concurrent use of these two vaccines. Thus, we excluded early recipients of Sinopharm (any vaccinated individuals who had achieved presumptive protection before January 30, 2021, the date when first individuals achieved presumptive protection after Pfizer/BioNtech vaccination). We found that the Pfizer/BioNtech vaccine was associated with statistically significantly fewer post-vaccination SARS-CoV-2 infections, hospitalisations, ICU admissions and deaths compared to the Sinopharm vaccine among those > 50 years of age. Similar findings were noted for those ≤ 50 years of age, although the difference in the death rates in this age group had low statistical significance which may be due to low numbers of events (Tables 1, 2, 3, 4). In a supplementary analysis, we compared the AZ/Covishield and Sputnik V vaccines in a similar manner using April 1, 2021 as the cutoff data. Again, the same trends were observed (Supplementary Table 1).

To examine whether individuals with co-morbid conditions were over-represented among recipients of a specific vaccine, we analyzed the prevalence of co-morbid conditions among the 148 vaccinated individuals who died (Table 5). While the limited number of deaths did not allow for statistical testing, we did not find obvious evidence of differences across the vaccines.

### Timeline of COVID-19 events

To determine how rates of COVID-19 infection, hospitalisation, ICU admission and death occurred over time, we constructed a time series for unvaccinated individuals and recipients of the four vaccines. Rates were calculated by dividing the number of outcomes (positive tests, hospitalisation/ICU admissions, or deaths) by the accumulated total number of people vaccinated (for each vaccine type—i.e., events per person at risk per day). This calculation was performed for each day to produce a time series. Unvaccinated rates were obtained by dividing the daily number of COVID-19 cases reported among such individuals by the total number of unvaccinated people in the population.

**Figure 3:**
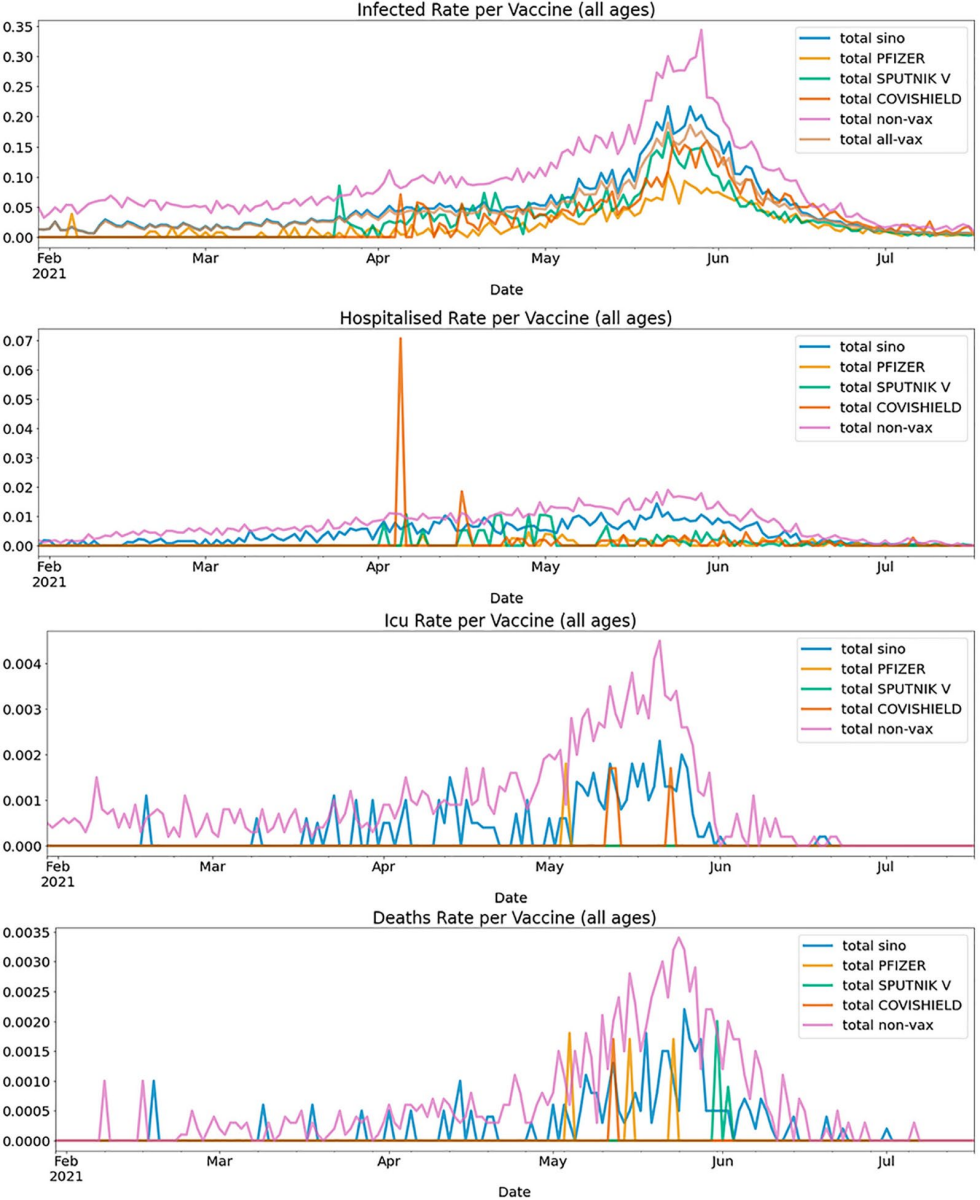
Timeline (events per person at risk per day) of infection, hospitalisations, ICU admissions and deaths for four vaccines compared to unvaccinated controls between Jan 30, 2021 to July 17, 2021.

We found a peak for all four clinical outcomes in the May/June 2021 period (Fig. 3), which corresponds to the rise in the Delta variant. As noted above, all four vaccines decreased the risk of infection, hospitalisation, ICU admission and death (Tables 1, 2, 3, 4, 5). However, the risk of ICU admission and deaths was significantly higher in the Sinopharm group compared to the Pfizer vaccine (3.31 fold for hospitalisation, and 7.39 fold for ICU admission for persons > 50 years of age). Other comparisons, with effect sizes and p values are presented in Tables 1, 2, 3, 4, 5.

**Figure 4:**
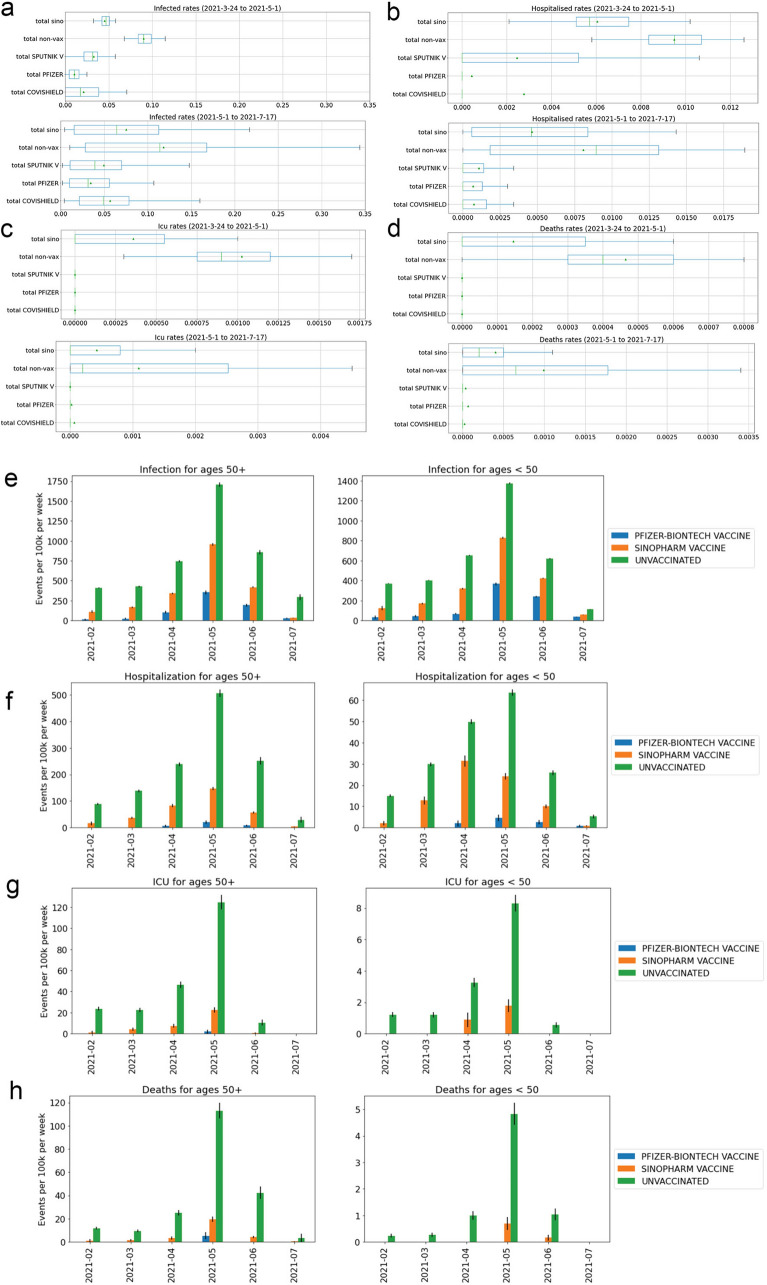
Comparison of infections, hospitalisations, ICU admissions, and deaths. (**a**–**d**) Box plots illustrating the distribution of rates for infections, hospitalisations, ICU admissions, and deaths. Where there is sufficient data, the whiskers illustrate the maximum and minimum, and boxes encompass the first and third quartiles. The median is displayed as a vertical line in the box. Data are split before and after May 1, 2021 when the Delta variant became prevalent in the studied population. (**e**–**h**) bar plots representing the events per 100,000 vaccine recipients from February 2021 to July 2021, comparing infections (**e**), hospitalisations (**f**), ICU admissions (**g**) and deaths (**h**). For (**e**–**h**), breakthrough infections after the Sinopharm and Pfizer vaccine are compared against unvaccinated individuals, divided by age cohorts > 50 years (left plot) and < 50 years (right plot). Standard error is shown as whiskers for each of these plots (**e**–**h**).

To formally compare the morbidity and mortality associated with post-vaccination infections for the various vaccines, and avoid multiple cross-comparisons, we restricted our comparison to examining the rates of post-vaccination infections causing hospitalisations, ICU admissions or death for the Pfizer/BioNtech and Sinopharm vaccine recipients. (Table 6). We found that the recipients of the Sinopharm vaccine were more likely to be hospitalized, be admitted to the ICU and die of COVIS-19 compared to recipients of the Pfizer/BioNtech vaccine, especially among those 50 years of age or older (Table 6). We also constructed a time series of post-vaccination infections, hospitalisations, ICU admissions and deaths disaggregated by age of the vaccine recipient (all years, and age < 50 and > 50 years) (Fig. 2 and Supplementary Fig. 1). Consistent with our prior findings, post-vaccination hospitalisations, ICU admissions and deaths were also highest among (Fig. 4) older Sinopharm vaccine recipients (> 50 years), with a peak during the May/June 2021 period, compared to younger recipients (age ≤ 50) and recipients of the other three vaccines.

### COVID-19 outcomes by age and sex

An analysis of hospitalisations and deaths for those > 60 compared to those < 60 years of age revealed similar trends as noted above for the ≤ 50 and > 50 year old group (Supplementary Table 2a and b). Using the Jan 30, 2021 cutoff, all four vaccines decreased hospitalisations among vaccines > 60 years by 1.83 fold for Sinopharm, 2.35 fold for Sputnik, 3.74 fold for Pfizer/BioNtech and 4.9 fold for AZ/Covishield). Similarly, using the April 1, 2021 cutoff, all four vaccines decreased hospitalisations among vaccines > 60 (by 2.43 fold for Sinopharm, 2.49 fold by Sputnik V, 5.40 fold for by Pfizer/BioNtech and 5.54 fold for AZ/Covishield between April 1 and July 17, 2021.

Similarly, an analysis of hospitalisation among men versus women recipients of the vaccines (Supplementary Table 3a and b) noted that all four vaccines reduced hospitalisation in men and women in a similar manner when the same rank order as noted above compared to unvaccinated individuals. In particular, with both the Jan 30 and April 1, 2021 cutoffs, Sinopharm was the least effective in both men and women in preventing post-vaccination COVID-19 related hospitalisations, followed by Sputnik V, Pfizer/BioNtech and AZ/Covishield. We noted that a smaller percentage of women received the AZ/Covishield vaccine (18.1% of AZ/ Covishield recipients were women versus 36.6% for Pfizer/BioNtech), likely related due to rare adverse effects (AE) reported among women who had received AZ/Covishield.

### COVID-19 outcomes among frontline workers and health care workers

Since frontline workers and health care workers (FLW/HCW) carry higher risks of SARS-CoV-2 infections^18,19^, we examined the risk of post-vaccination infections in this population (Supplementary Table 4). A total of 5,784 FLW and HCW workers were vaccinated, representing approximately 89 percent of the documented FLW and HCW population in Bahrain. Of the total vaccinated population in the country, 0.59% of HCW/FLW received the Sinopharm, 0.75% with Pfizer/BioNtech, 0.32% with Sputnik V and 1.07% with AZ/Covishield vaccines. The ratio of the percentage of vaccinated FLW/HCW compared to the percentage of the overall population vaccinated for Sinopharm and for Pfizer/BionTech vaccines was nearly 1; in contrast, the ratio was 1.86 for AZ/Covishield, and 0.57 for Sputnik V, indicating that a relatively larger proportion of FLW/HCWs received the AZ/Covishield and a lower proportion received the Sputnik V vaccines compared to the general population.

Post-vaccination SARS-CoV-2 infections, hospitalisations, ICU admissions and deaths among FLW/HCW are shown in Supplementary Table 4. Since FLW/HCW were given the highest priority in vaccination efforts, these data do not exclude any of the vaccines, including the earliest vaccine recipients. The highest rate of post-vaccination SARS-CoV-2 infection was observed in Sinopharm vaccinated individuals (11.7%) followed by the AZ/Covishield group (10.24%). The comparatively high risk of infection in the AZ/Covishield vaccine may be because its deployment was co-incident with the rise in the Delta wave; however, clinical outcomes in this cohort were very mild, with 1 hospitalisation and no deaths. Hospitalisations and ICU admissions were the highest in the Sinopharm groups. Two deaths occurred among FLW/HCW, both among Sinopharm recipients (Supplementary Table 4). A statistical comparison of deaths between vaccine groups is not possible given the low numbers.

**Figure 5:**
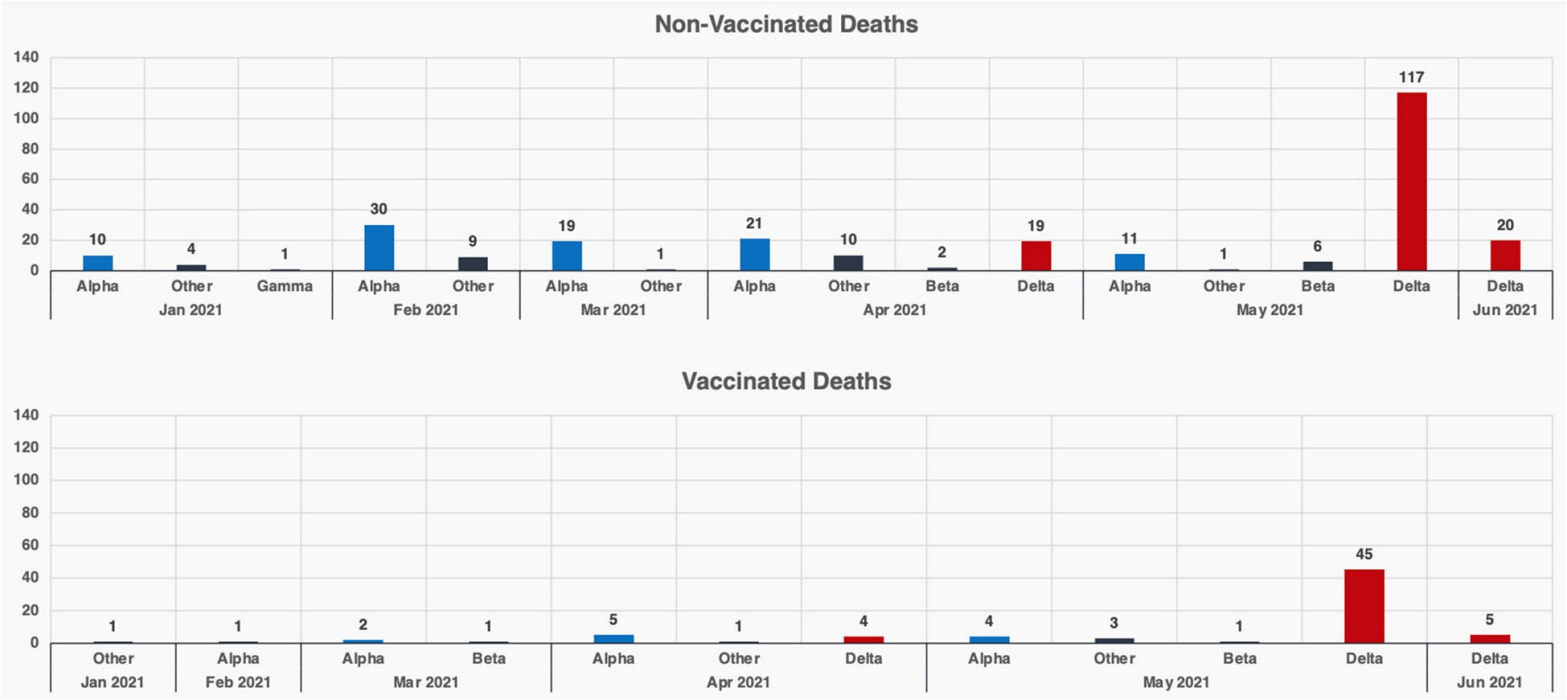
Variant sequencing for SARS-Cov2 infected individuals who died (presumptively from SARS-Cov2 infection). Variants detected between January 1 2021 to June 30 2021 are shown in the following brackets: Alpha (B1.1.7), Beta (1.351), Gamma (B.1.28.1) and Delta (B.1.617.1 and B.1.617.2) and Other for vaccinated and unvaccinated individuals.

Sequencing was performed for all SARS-CoV-2 isolates from individuals who died from COVID-19 after January 1, 2021. As shown in Fig. 5, prior to May 2021, hospitalisations and deaths were dominated by unvaccinated individuals infected predominantly by the Alpha variant. After May 2021, the number of deaths began to rise in the vaccinated population, although still fewer deaths reported compared to among the unvaccinated group, and both unvaccinated and vaccinated deaths were dominated by individuals infected with the Delta variant.

### Durability of vaccine protection

To assess the possibility of waning of protection since vaccination, we determined the percent of COVID-19 cases that were identified during June and July 2021 and that occurred among individuals who had been vaccinated and achieved PP in overlapping two-month periods (e.g., January through February, February through March, March through April 2021 by vaccine (Supplementary Fig. 2).

The pattern in the proportion of June 2021 COVID-19 cases, based on the 2-month period of achieving PP by vaccine, does not indicate an increasing trend, as would be the case if waning protection was occurring in this cohort. For instance, the highest fraction of post-vaccination SARS-CoV-2 infections for Sinopharm vaccine recipients was observed in the cohort that received vaccines in March through April, April through May, and May through June. A similar trend was observed for the 3 other vaccines; however, because other vaccines (e.g., AZ/Covishield) were rolled out later, there was a limited number of cases reported among those vaccinated in earlier months.

However, when we restricted this analysis to individuals > 60 years of age, we observed a decrease in post-vaccination SARS-CoV-2 infections in more recently vaccinated individuals (e.g., the rate of infections in Pfizer/BioNtech vaccines fell from 0.20 in March–April period to 0.025 in the May–June period. (Supplementary Fig. 2). The exception to this trend was the Sinopharm vaccine, which showed a rise in infection rates up to March–April and then fell subsequently.

### Comparison of hospitalisations among Bahrainis versus non-Bahrainis

We compared the hospitalisation rates (%) between PCR + Bahrainis and non-Bahraini registrants in the ISIEH database. Tourists are excluded. Rather “non-Bahrainis” represent workers who live in Bahrain, have residency certificates, and equal and free access to vaccination, medical care and hospitalisation. Notably, across all vaccines, we found a higher percentage of hospitalisation in Bahrainis compared to non Bahrainis (effect size: Sinopharm; Pfizer; Covishield and Sputnik. All p values comparing Bahrainis and non-Bahrainis were < 0.01 (Supplementary Table 5). To understand the effect, we plotted a histogram of ages of PCR positive Bahrani and non-Bahrainis (Supplementary Fig. 3). Notably, the Bahraini population was represented as a bimodal curve, with two populations—one younger, and a second older population. We discuss this bimodal curve, and its possible explanation of the increased hospitalisation of PCR positive cases below.

## Discussion

The COVID-19 vaccination program in Bahrain, and the high frequency of SARS-CoV-2 testing combined with its detailed individual case monitoring allowed for the opportunity to examine the real-world relative protection of the four vaccines used, Sinopharm, Covishield, Pfizer/BioNtech and Sputnik V, before and during the dominance of the Delta variant. Compared to unvaccinated individuals, the four vaccines were all highly effective in reducing the frequency of post-vaccination outcomes of SARS-CoV-2 infections, hospitalisations, ICU admissions and death, prior to, and during the period when the Delta variant predominated in the country. However, compared to the three other vaccines, individuals vaccinated with Sinopharm vaccine had a higher risk of all post-vaccination outcomes except death, especially among older individuals. These “real world” findings may inform policy decisions about vaccine choices and anticipated effects.

Targeted sequencing of virus isolates from individuals who died from COVID-19 showed that deaths from January through April 2021 were dominated by the Alpha variant, as the most dominant variant in the country at that time. From May 2021 onwards, the total fraction of infections by Delta rose from 30 to 90 percent by July 2021. Deaths associated with the emergence of the Delta variant also rose in May 2021, identified in 86 percent and 84 percent of the deaths reported for unvaccinated and vaccinated individuals, respectively. In June 2021 all deaths in both groups were caused by the Delta variant.

We did not find evidence to support a waning of immunity. However, noting the increased risk in those > 60 years of age, there was a modest signal consistent with waning protection among the older (> 60 y) with the Pfizer vaccine recipients prior to the dominance of the Delta variant. There are two competing hypotheses concerning our observation of the fall of post vaccination SARS-CoV-2 infections in > 60 year old individuals in association with timing of vaccination. First, it may be due to the fact that hospitalisations caused by the Delta surge in Bahrain peaked and fell exactly as the post-vaccination infections peaked and fell—i.e., in the months of May and June 2021 (Fig. 2). Thus, the falling rates of post-vaccination SARS-CoV-2 infections may reflect the overall decrease in post-vaccination infections overall. Conversely, the decreased infection rates in those recently vaccinated may truly indicate waning protection against the Delta strains. Our data do not allow us to distinguish between these two hypotheses.

Given the infeasibility of a randomized study directly comparing the efficacy of four vaccines to an unvaccinated comparator arm, it becomes particularly important to seek insights from observational data while making every effort to avoid potential biases and confounders. For example, we excluded from analyses, those who were early recipients of the Sinopharm and Pfizer/BioNtech vaccines in order to avoid a potential bias as early vaccination efforts focused on older individuals and FLW/HCW. To address the confounding effects of age and sex, we stratified outcomes by two age cutoffs, age 50 and age 60, as well as sex.

We also analyzed outcomes separately for FLW/HCW who received Pfizer/BioNtech and Sinopharm vaccinations in proportions similar to the overall vaccinated population, thus the higher rates of post-vaccination COVID-19 related outcomes in the Sinopharm group was not driven by higher uptake of that vaccine by this population.

Unfortunately, it is not possible to perform detailed cohort by cohort analysis of ages > 70, 80 and 90 with adequate statistical power on this dataset. For instance, for the Pfizer vaccine, there are only 17 individuals of age > 70, and 1 individual of age > 80 that had post vaccination breakthrough infection after Feb 1. In the age > 70 cohort, there were only 5 admissions and 1 ICU admission, and 1 death (in a 76-year-old-male) in the Pfizer vaccinated group. The average age of infected individuals who are > 70 in this group is 75. In comparison, there are 454 post vaccination infections in the Sinopharm group. Of these, 105 were above 80 years of age. There were 10 deaths in this group (age > 80). The average age of post vaccinee breakthrough infections in those of age > 70 was 77.03. These numbers are not powered for statistical analysis. For instance, for post-vaccination breakthrough infection, the sample size is 105 vs 1 (Sinopharm vs Pfizer) in the > 80 years group for infection, and deaths of 10 versus 0 (Sinopharm vs Pfizer).

We performed another analysis. Using hospitalization after breakthrough infection as an adverse outcome for all ages, and Jan 30th as a cutoff date (when Pfizer and Sinopharm were both available), we find that the 620 Sinopharm vaccinees were hospitalized (n = 620). The average age = 56.74 and the proportion of age > 70 = 0.20. For Pfizer: n = 19, average age = 56.47, proportion > 70 = 0.26. The p value for Sinopharm vs Pfizer in this dataset in 0.9, and proportions are nearly identical. Therefore, we do not think that this confounds the analysis significantly.

We acknowledge this as a limitation of the data and believe that these numbers simply reflect the greater use of the Sinopharm vaccine compared to the Pfizer vaccine. Therefore, we used an age cutoff of > 60 to analyze the data (Supplementary Table 2).

Our study is limited by the absence of socioeconomic status information and thus, we cannot exclude potential confounding by such factors. We are limited also in our ability to account for the variabilities in the quality and accessibility of health care access. However, we note that health care access is free and universal across Bahrain. The study also has several strengths, including the detailed clinical outcomes for the whole population of the country, with outcomes missing on only 1.17% of the entire population. In addition, while SARS-CoV-2 testing was not exclusively done at random, testing frequency was very high, with Bahrain being one of the top ten countries globally in terms of tests administered per population.

We note that the Omicron strain has become the dominant COVID strain across the globe but the Delta still prevails in some parts (e.g., in France, 80 percent of SARS-Cov2 strains were Omicron in December 2021; data for January is still being compiled^20^). Despite the prevalence of Omicron, we feel that systematic maintenance of a national database as in Bahrain, the methods used to determine and quantify breakthrough infections over time, and observational studies (despite their confounding effects) provide a model by which breakthrough infections caused by Omicron (and future strains) and their dependence on the initial vaccine provided. This highlights the continued value of this study.

We conclude that COVID-19 vaccination is highly effective at protection against COVID-19 and related key outcomes, especially among individuals older than 50 years of age. However, the performance of the Pfizer/BioNtech vaccine was found to be superior to the Sinopharm vaccine, with the latter associated with a higher risk for post-vaccination infections, hospitalisations, ICU admissions and deaths, especially among those > 50 years of age, and in the context of emergence of the Delta variant of SARS-Cov2.

## Methods

### Vaccination program in Bahrain

The Kingdom of Bahrain has introduced a series of measures to ensure equal access to the vaccines regardless of race, age, nationality, gender, or income. Vaccines were administered at no cost. Thirty-one vaccination sites were established in the country to ensure access. Protocols were also available for registration, counselling/consultation vaccination and observation. The sites were geographically distributed at 27 primary care centers, The International Exhibition and Convention Centre, Sitra Mall, King Hamad University Hospital and Bahrain Defense Force Hospital mobile vaccination units were utilized to provide the vaccine to senior citizens, persons with special needs, inmates, and others who are unable to receive the vaccine at one of the designated vaccination centers.

The national vaccination for the general population started on December 16, 2020 through 31 vaccination centers. Initially, high risk populations (i.e., frontline workers, individuals > 60 years of age, and patients with morbid obesity, any immunocompromised state, diabetes, cardiopulmonary disease and hypertension) were given priority when booking appointments over the general population. By December 16, 2020, vaccination was extended to the general population. Four COVID-19 vaccines were used. The Sinopharm vaccine was deployed first, in part because Bahrain was involved in clinical trials with Sinopharm, followed by Pfizer/BioNtech, Sputnik V and AZ-Covishield. Note that the early cohort of Sinopharm vaccines have been censored.

This study's protocol was reviewed and approved by the National COVID-19 Research Committee in Bahrain (Study ID: CRT-COVID2021-154).

All procedures performed in this study involving human participants were in accordance with the ethical standards of the National COVID-19 Research committee and with the 1964 Helsinki Declaration and its later amendments or comparable ethical standards.

This study does not contain any studies involving animals performed by any of the authors.

All procedures performed in this study involving human participants were in accordance with the ethical standards of the National COVID-19 Research committee and with the 1964 Helsinki Declaration and its later amendments or comparable ethical standards.

This study does not contain any studies involving animals performed by any of the authors.

Waiver of documentation of informed consent was approved by National COVID-19 Research committee in Bahrain. All criteria met for a Waiver of Consent as per national research ethics and compliance.

### Data collection database

A country specific Information system (ISEHA) has all medical history of individuals and is centralized and used for all covid related health information. Anonymized electronic health records were retrieved for the study period December 9, 2020—July 17, 2021.These records include: (a) Patient demographics, a random ID used to link records, year of birth, sex, coded geographical location of residence, nationality and chronic comorbidities including: cardiovascular disease, type 2 diabetes, high blood pressure, immunosuppression, high BMI, chronic kidney disease (CKD) and chronic obstructive pulmonary disease (COPD).

### Identification of positive cases and variants

All cases were diagnosed based on RT-PCR tests of nasopharyngeal samples. The majority of RT-PCR tests were conducted using Thermo Fisher Scientific (Waltham, MA) TaqPath 1-Step RT-qPCR Master Mix, CG (catalog number A15299) on the Applied Biosystems (Foster City, CA) 7500 Fast Dx RealTime PCR Instrument. The assay used followed the World Health Organization protocol and targeted the E gene^21^. If positive, the sample was confirmed by RdRP and N genes. The E gene CT value was reported and used in this study. CT Values < 37 were considered positive. Whole genome sequencing was used to identify the common variants of concerns using Illumina/ARTIC and COVID-Seq protocols. The data were analysed with the Abiomix platform. Sequencing was undertaken at the national COVID-19 Molecular public health laboratory where all the samples get tested there. Spike gene target status on PCR was used as a second approach for identifying each variant.

### Statistical analysis

Statistical analysis was performed as follows. Analysis was performed using statsmodels 0.10.2. Positive events were filtered to be either from persons of 18 years old or older and to unvaccinated individuals (once vaccinated count as this) or from individuals who were fully vaccinated on or after 2021-01-30. Counts of who is at risk in each subgroup, for the purpose of rate denominators, reflect available data (unvaccinated) or all individuals (living or dead) who were fully vaccinated after this date.

#### Odds ratio analysis

Event rate comparisons were made in terms of odds ratios (for hospitalisation, ICU, death) using Cochran Mantel Haenszel analysis of event counts stratified by day. The first analyses were made using the full study period comparing Pfizer to Sinopharm and unvaccinated to Sinopharm. Similar analyses were made when appropriately small p-values (< 0.001) were found in the first analysis for the data broken by month or broken by before/after 2021-05-01.

#### Event rate displays

To display event rates the computation for a given time period went as follows: (1) Compute daily rates (i.e. event count in a given day/ at risk count for that day). (2) Sum the daily rates over the time period. (3) Convert this figure to the units of per 100 K persons and per 7 days, taking account of the number of days in the time period. Similar calculations were made for the variance of this quantity using the delta method and assuming that the count variance on each day is an independent Poisson (given the true rate parameter), that is plugging in the observed count as a variance for the count and propagating this to compute a variance for the computed rate. This was square-rooted to give a standard error for the plots.

#### PCR Positive event proportion analysis

To give confidence intervals to the proportions of events, Jeffreys intervals were used using an alpha value of 0.01. In this way, when considering one event outcome at a time, the five vaccine conditions can be considered to have simultaneous confidence intervals at 0.05-level (i.e. the FWER is controlled). For formal pairwise comparisons between vaccination conditions of such a rate, the five all-pairs two-by-two tables of event/non-event vs vaccine-group1/vaccine-group2 were analyzed using Fisher exact p-values. The resulting 10 p-values were converted to corrected p-values using the Holm-Sidak method.

#### Calculations for infection rates and odds ratios

Rates of infection were calculated using the same method as described under “Event rate displays” (above). As with event rates, infection rate per week was calculated by dividing the number infected by the number at risk per month and then normalizing to 100 K people. The infection rate over the full period of the study is the weighted average of the monthly infection rates where the weights are proportional to the number of days in each month in the study period. Odds Ratios were calculated using the Cochran Mantel Haenszel analysis of event counts stratified by day (see “Odds Ratio Analysis” above). In other words, the infection rate over the full period of the study is the weighted average of the monthly infection rates where the weights are proportional to the number of days in each month in the study period.

## Supplementary Information

Supplementary Information.

## Data Availability

The datasets analysed in the current study are not publicly available because they belong to Bahrain government but are available from the corresponding author on reasonable request.
