# Supplementary material for: Post-vaccination outcomes in association with four COVID-19 vaccines in the Kingdom of Bahrain
Source: Sci Rep. 2022 Jun 2;12:9236. doi: 10.1038/s41598-022-12543-4 (PMC9161761; doi:10.1038/s41598-022-12543-4)
Supplement: Supplementary file 1 — Supplementary Information. [file 41598_2022_12543_MOESM1_ESM.docx]

Post-vaccination Outcomes in Association with Four COVID-19 Vaccines in the Kingdom of Bahrain

*Authors: Manaf AlQahtani ^1^, Xing Du ^2^, Sujoy Bhattacharyya ^3^, Abdulla Alawadi ^1^, Hamad Al Mahmeed ^1^, Jaleela Al Sayed ^1^, Jessica Justman ^4^, Wafaa M. El-Sadr ^4^, Jack Hidary ^5^ and Siddhartha Mukherjee ^3^*

**Supplementary data**

**Supp Fig 1a, Infection**

**
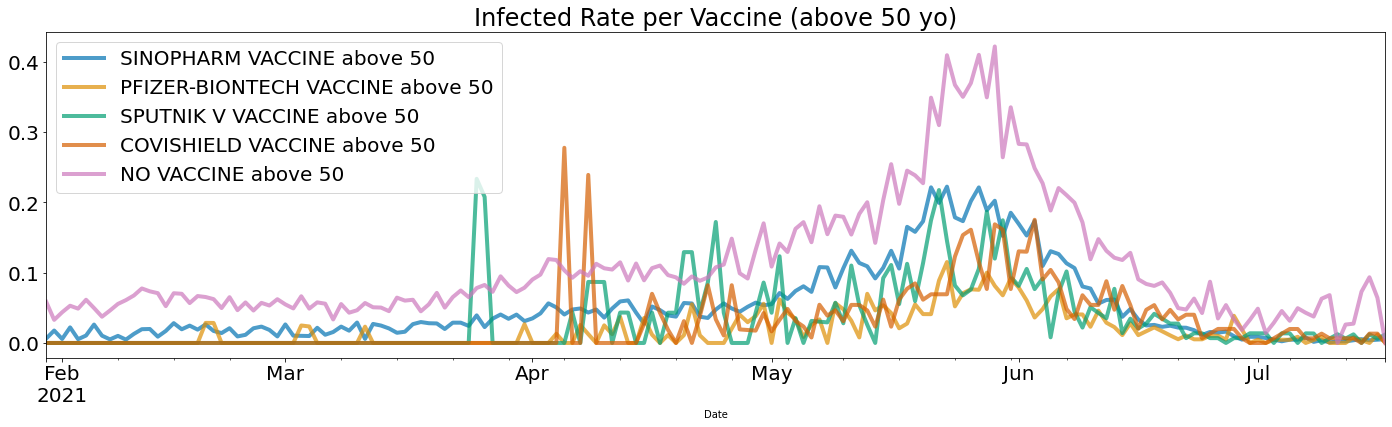
**

**
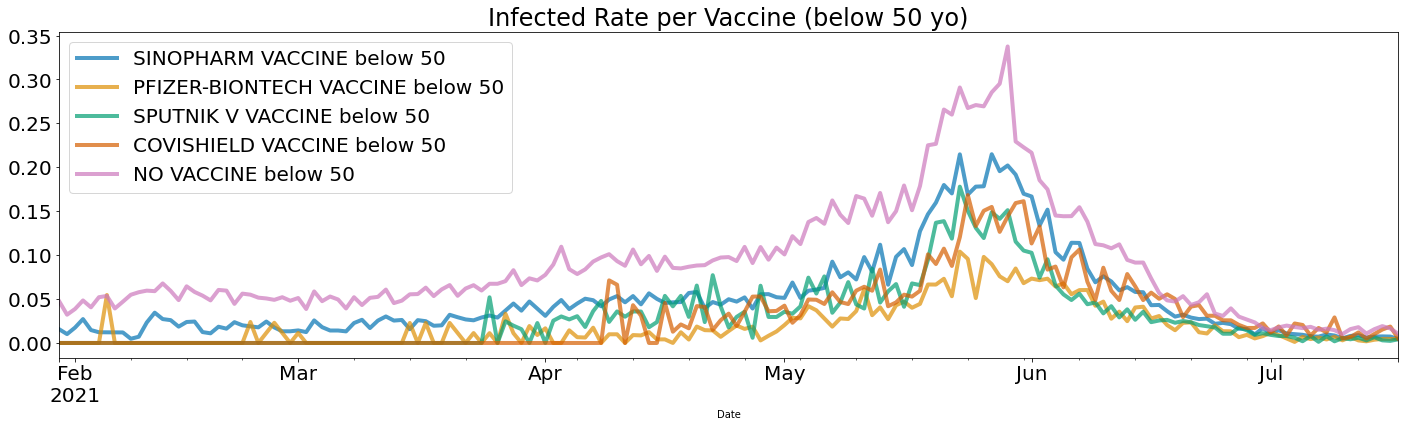
**

**Supp Fig 1b, Hospitalisation**

**
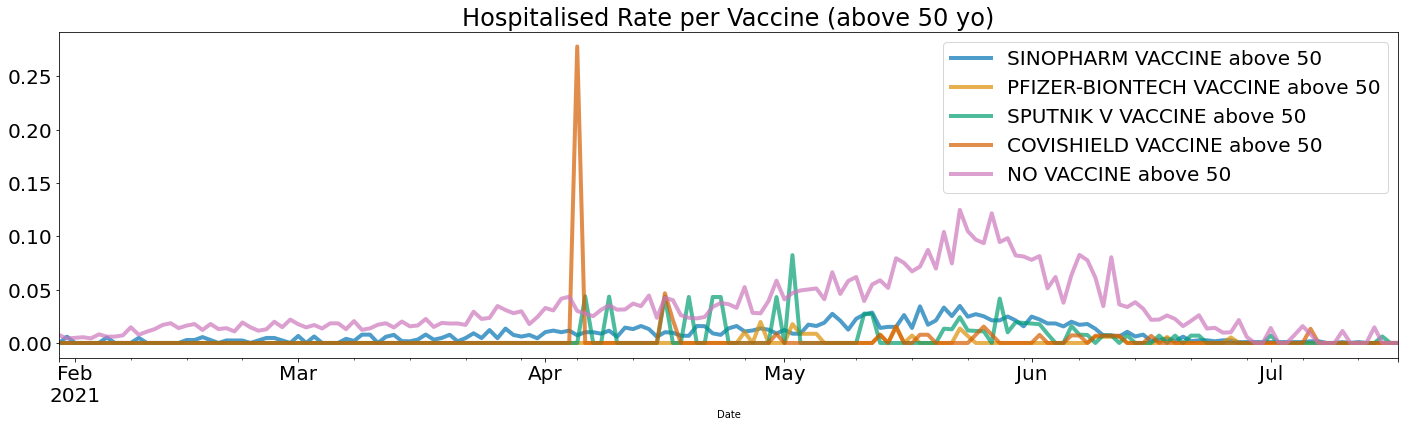
**

**
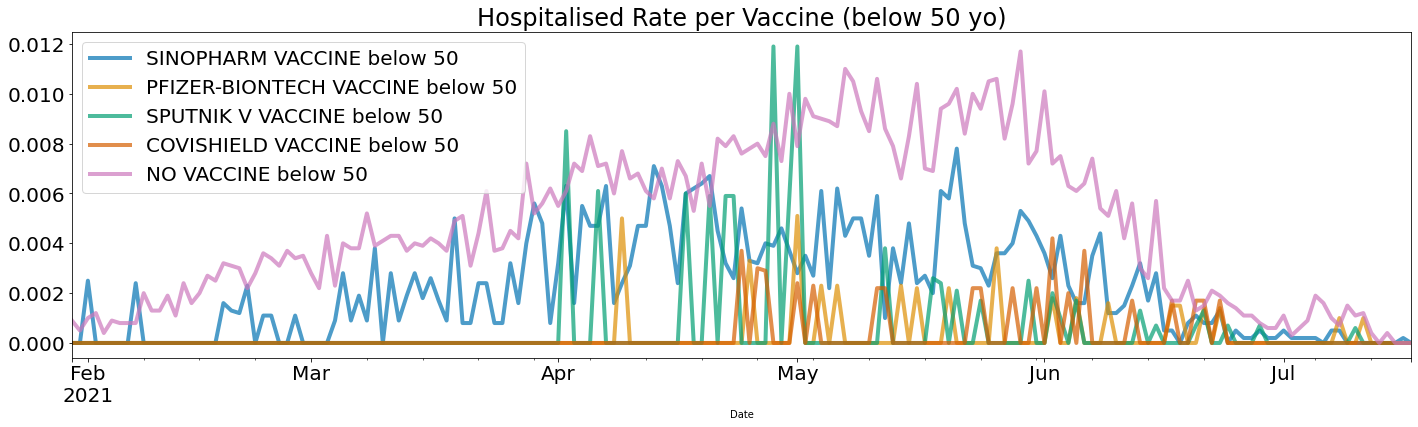
**

**Supp Fig 1c, ICU Admissions**


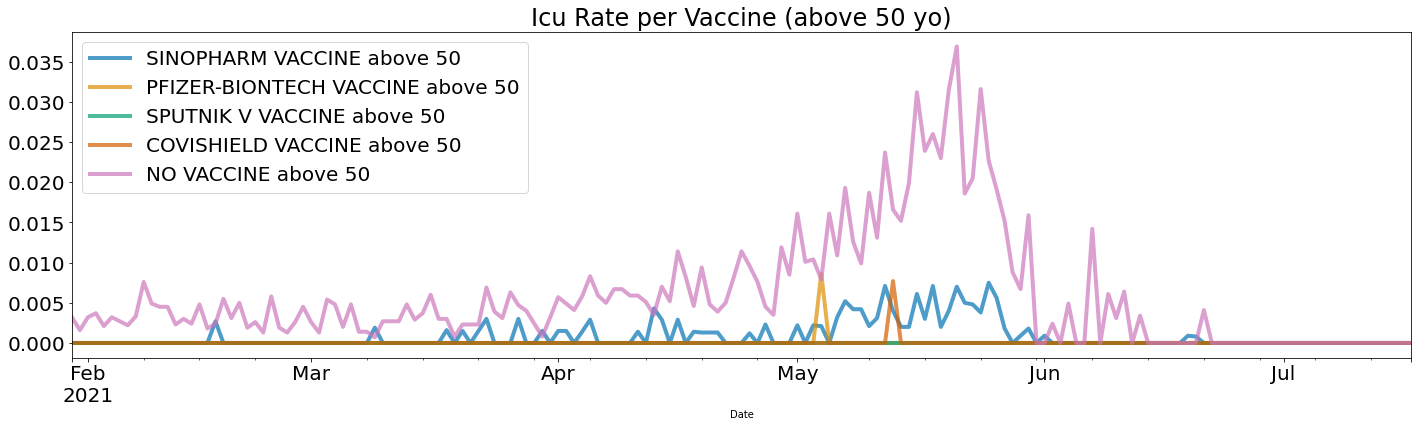


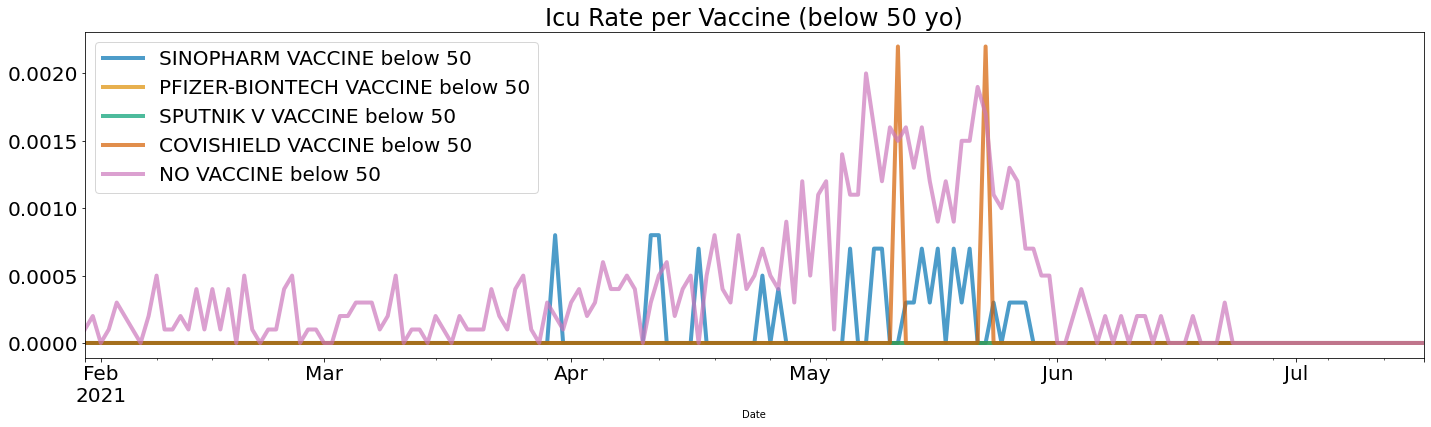


**Supp Fig 1d, Deaths**


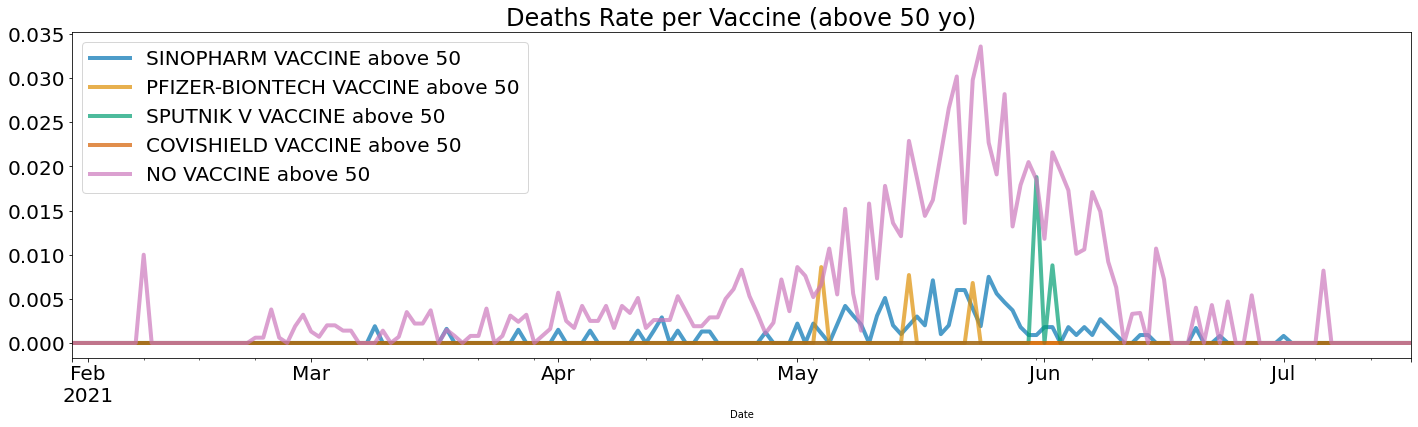


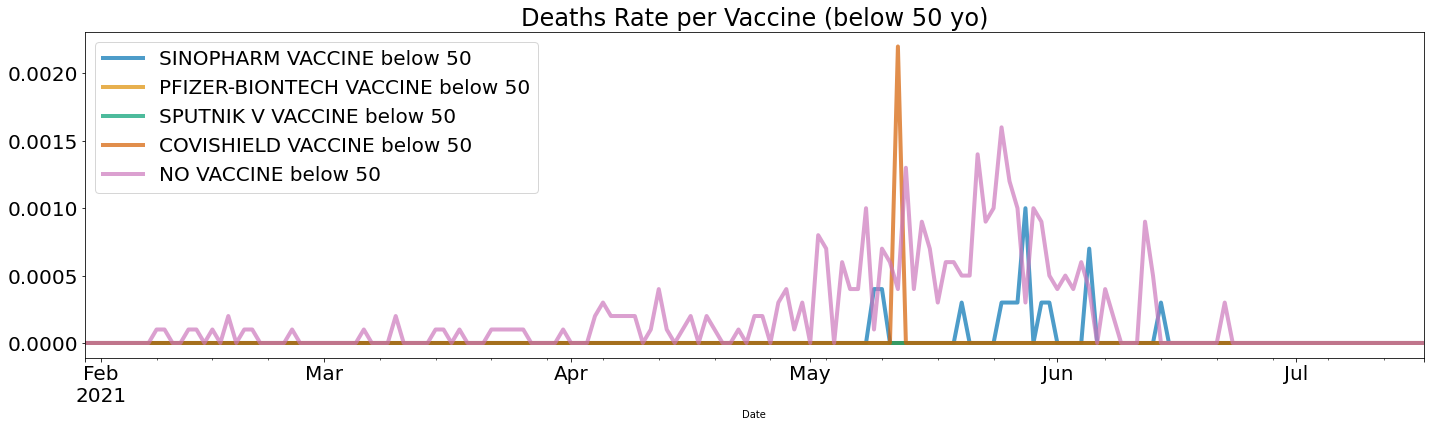


***Supplementary Figure 1,* Outcomes Rates among vaccinated and unvaccinated individuals by age cohorts >50 and <50 years.** Time series illustrating rates of a) Infections, b) Hospitalisations, c) ICU admissions, and d) deaths for all vaccines and unvaccinated for the above and below 50 age cohort. Events per 100k population per week are also shown as a time series where a maximum is seen in the month of May (when Delta became the dominant variant).

**Supp Fig 2a, All Ages
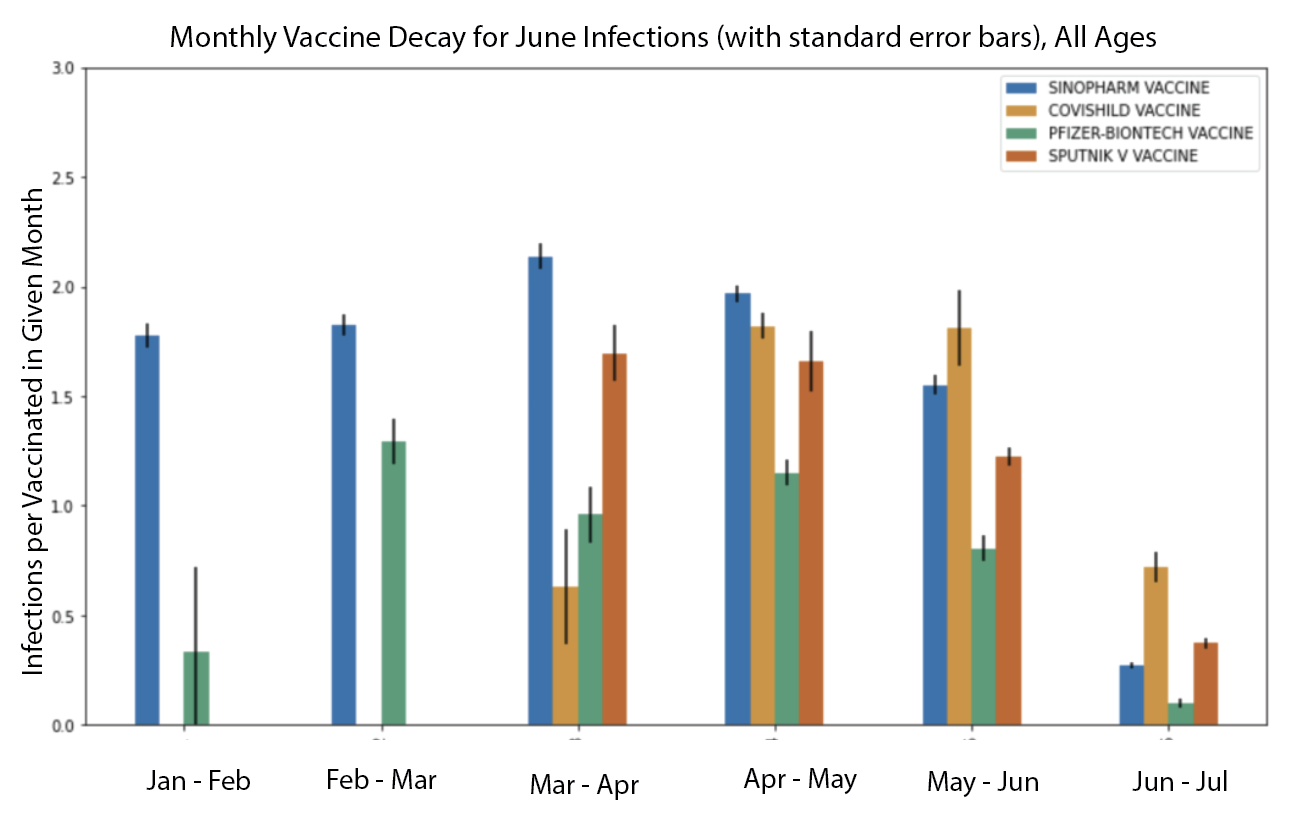
**

**Supp Fig 2b, Over 60 years old**

***
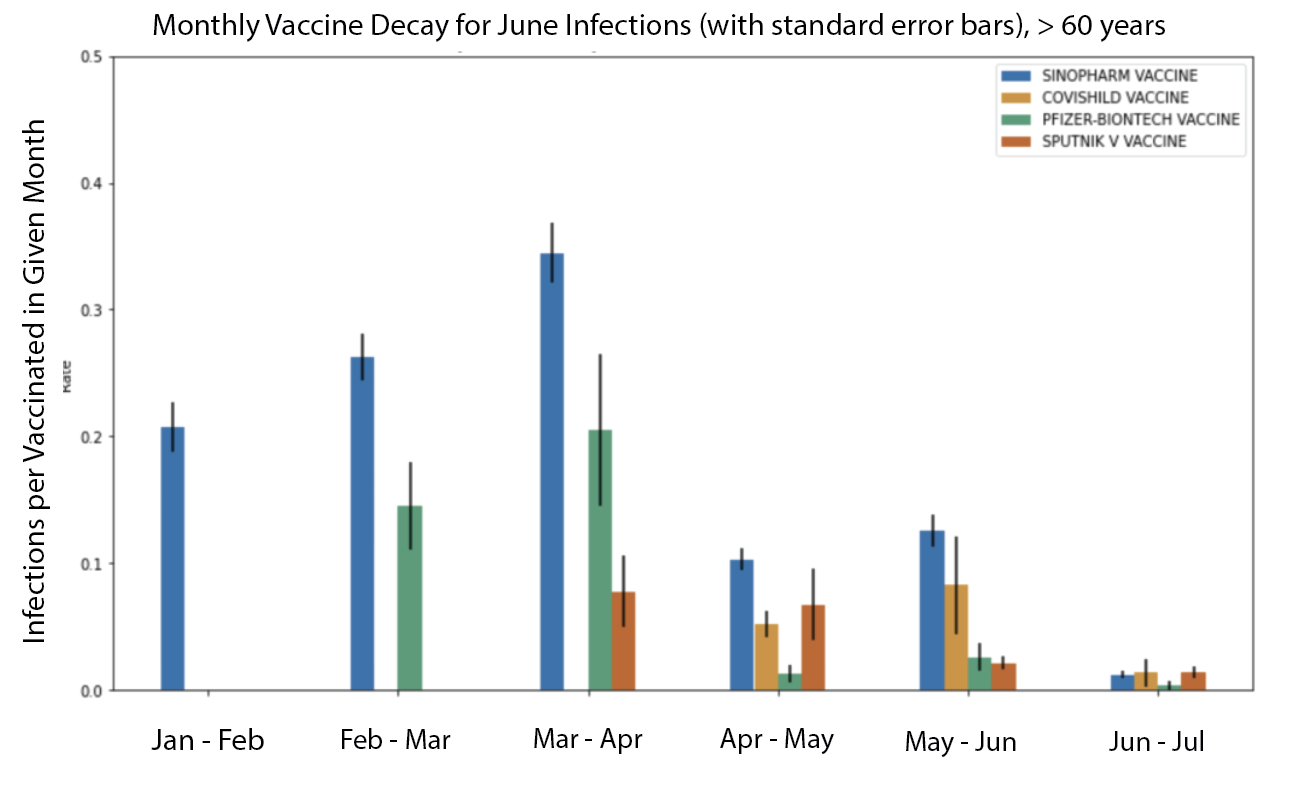
***

**Supplementary Figure 2 (a, b): Post Vaccination Infections in June, based on vaccinations from January 2021 through July 2021.** The y axis shows the rates of infections in June of individuals vaccinated and with presumptive protection (14 days after second dose) in earlier months (Jan-Feb etc).  These data did not eliminate early recipients of vaccines (hence no bars for Pfizer, Covishield and Sputnik V in Jan-Feb). Error bars represent the standard error.

A reduction in rate from (March-April) to (Apr - May) was seen in Sinopharm (OR 11.9, p < 0.001), and Pfizer-BioNTech (6.4, p < 0.001).


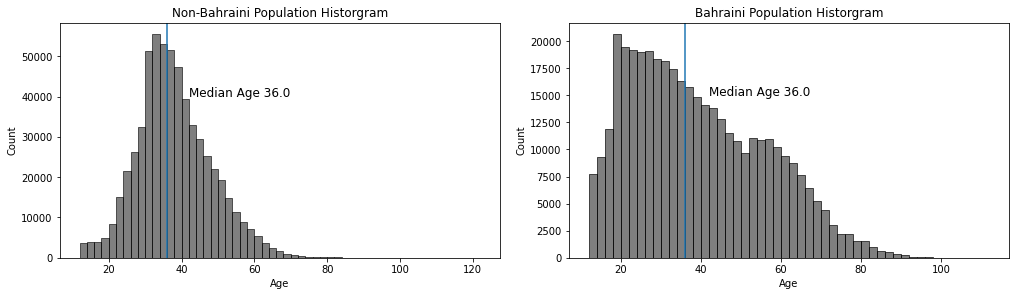


**Supplementary Figure 3, Histograms by age of Bahraini and non-Bahraini individuals tested positive by PCR*.*** While the median ages were identical, the distributions show clear differences. Note that 60.3% of the total positive-tested population are non-Bahraini. However, Bahrainis represent a bimodal population with younger residents, as well as older residents.

Supp Table 1a

|  | **Covishield vs Sinopharm** | | | | | | | | | | | |
| --- | --- | --- | --- | --- | --- | --- | --- | --- | --- | --- | --- | --- |
|  | ALL | | | | Over 50 | | | | Under 50 | | | |
|  | Rate (Covishield) | Rate (Sino) | p | OR | Rate (Covishield) | Rate (Sino) | p | OR | Rate (Covishield) | Rate (Sino) | p | OR |
| **Infections** | 336.13 | 462.97 | <.001 | 0.78 | 321.68 | 490.17 | <.001 | 0.65 | 340.06 | 452.94 | <.001 | 0.82 |
| **Hospitalisations** | 10.98 | 35.60 | <.001 | 0.19 | 31.93 | 81.60 | <.001 | 0.18 | 2.973 | 18.584 | <.001 | 0.23 |
| **ICU admissions** | 0.33 | 2.93 | <.001 | 0.15 | 0.50 | 8.70 | <.001 | 0.07 | 0.28 | 0.75 | **NS**  **0.35** | 0.51 |
| **Deaths** | 0.11 | 2.21 | <.001 | 0.06 | 0.0 | 7.67 | <.001 | 0.0 | 0.14 | 0.24 | **NS**  **0.57** | 0.57 |

Supp Table 1b

|  | **Sputnik V vs Sinopharm** | | | | | | | | | | | |
| --- | --- | --- | --- | --- | --- | --- | --- | --- | --- | --- | --- | --- |
|  | ALL | | | | Over 50 | | | | Under 50 | | | |
|  | Rate (Sputnik) | Rate (Sino) | p | OR | Rate (Sputnik) | Rate (Sino) | p | OR | Rate (Sputnik) | Rate (Sino) | p | OR |
| **Infections** | 317.88 | 462.97 | <.001 | 0.66 | 328.80 | 490.17 | <.001 | 0.67 | 316.77 | 452.94 | <.001 | 0.66 |
| **Hospitalisations** | 10.99 | 35.60 | <.001 | 0.26 | 48.66 | 81.60 | <.001 | 0.55 | 6.06 | 18.584 | <.001 | 0.26 |
| **ICU admissions** | 0.0 | 2.93 | <.001 | 0.0 | 0.0 | 8.70 | **<.05** | 0.0 | 0.0 | 0.75 | **NS**  **0.05** | 0.0 |
| **Deaths** | 0.19 | 2.21 | <.001 | 0.14 | 1.79 | 7.67 | **NS** | 0.36 | 0.0 | 0.24 | **NS**  **0.1** | 0.0 |

**Supplementary Table 1, rate comparison (events per 100,000 persons per week) between Covishield with Sinopharm and Sputnik V with Sinopharm (using April 1 Cutoff).** Rate comparisons of the Sinopharm vaccine against AZ/Covishield (a) and Sputnik V (b) with Rates, Odds Ratios and p values for the population over 50 and under 50. The four parameters analyzed were infections, hospitalisations, ICU admissions and deaths, p values are reported as <0.001, <0.05 and NS if low significant. Note that early vaccines of Sinopharm between Dec 9th and April 1, 2021 were eliminated because AZ/Covishield and Sputnik vaccinations began on April 1 2021.

***Supp Table 2a: Hospitalisation (%) of SARS-COV-2 PCR positive cases in ages above and below 60 years with an Jan 1 cutoff date.***

***
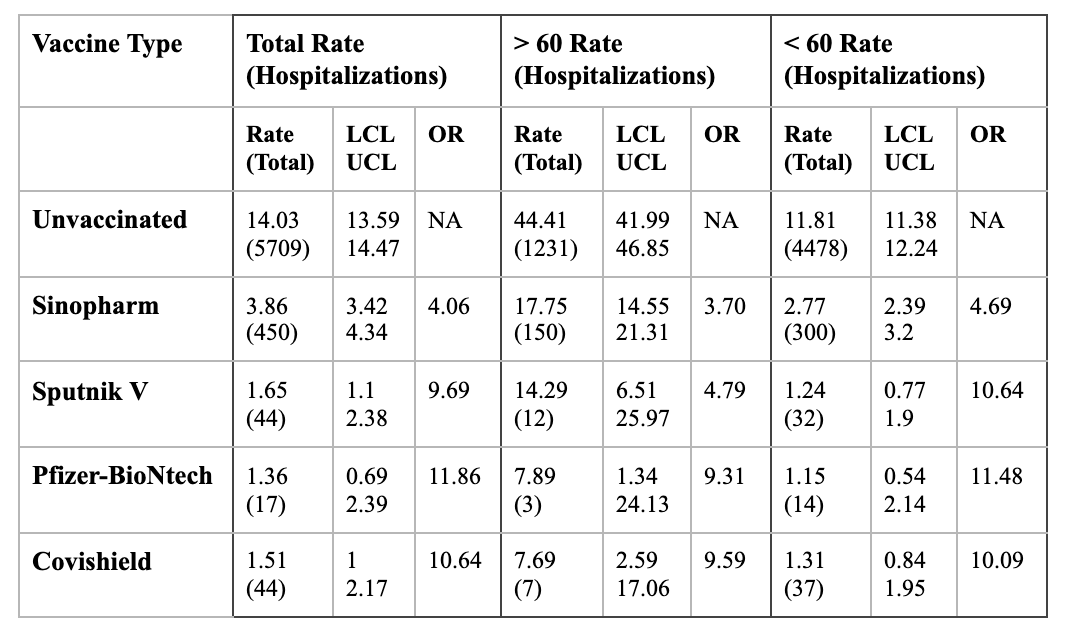
***

***p-values for all comparison are <0.001***

***Supp Table 2b: Death (%) of SARS-COV-2 PCR positive cases in ages above and below 60 years with an April 1 cutoff date.***

***
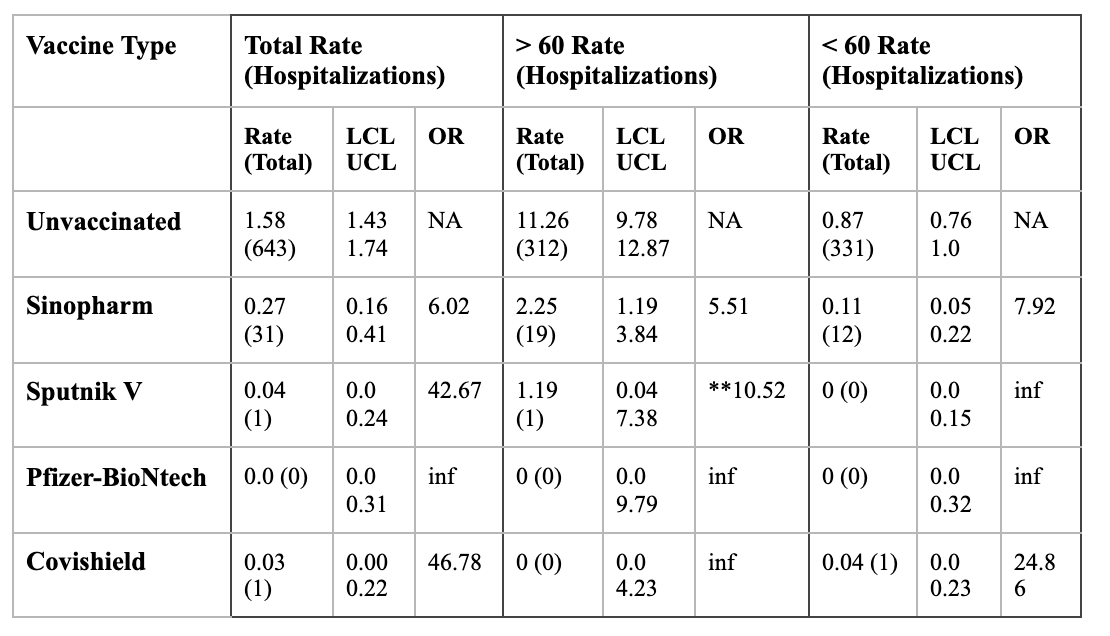
***

***p-values for all comparison are <0.001, except for OR marked with ** (p-value < 0.05).***

***Supp Table 3a Hospitalisations (%) of Males versus Females (18+ ages), with Odds Ratios (OR) for SARS-Cov2 positive cases among Unvaccinated, and Sinopharm, Sputnik V, Pfizer-BioNtech and Covishield vaccinated individuals with a Jan 30th cutoff date. p-values for all pairwise comparisons with the unvaccinated cohort are <0.001***


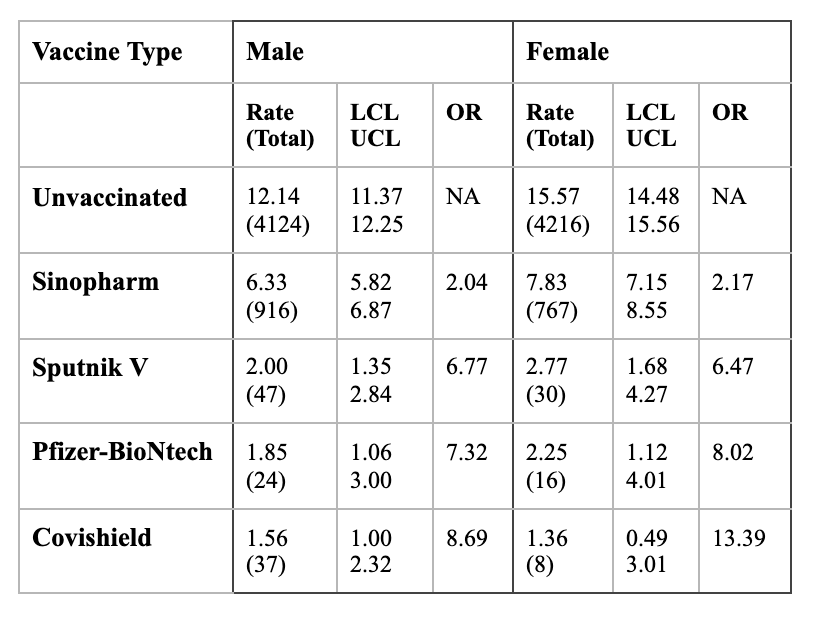


***Supp Table 3b Hospitalisations (%) of Males versus Females (18+ ages), with Odds Ratios (OR) for SARS-Cov2 positive cases among Unvaccinated, and Sinopharm, Sputnik V, Pfizer-BioNtech and Covishield vaccinated individuals with an April 1 cutoff date.***

***
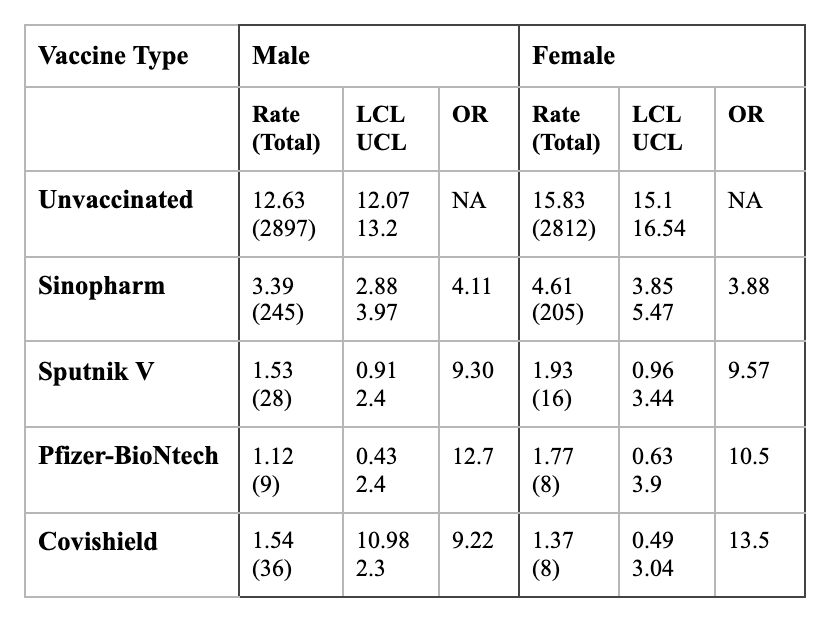
***

***Supplementary Table 3: Hospitalisation rates for SARS-Cov2 positive cases in males and females. Note that a January 30th cutoff was used in (a) and April 1 cutoff in (b), excluding early recipients of the Sinopharm vaccine (Pfizer vaccination was deployed on Jan 30n AZ/Covishield and Sputnik V vaccines were deployed on April 1). All p values for comparison to Sinopharm were <0.001***

| **Vaccine Type** | **Infected (Total Vaccinated); Rate % of infected among vaccinated.** | **HCW, FLW / Total Vaccinated Population** | **Comparison to deployment in total population (1 = identical)** | **Total Hospitalisations; Rate [LCL, UCL]** | **Total ICU, Rate [LCL, UCL]** | **Total Deaths, Rate [LCL, UCL]** |
| --- | --- | --- | --- | --- | --- | --- |
| **Sinopharm** | 393 (3358); 11.7% | 0.59% | 1.02 | 23; 5.85 [3.32, 9.44] | 5; 1.27 [0.33, 3.37] | 2; 0.50 [0.05, 2.11] |
| **Pfizer** | 19 (1027); 1.85% | 0.75% | 1.01 | 0; 0.00 [0.00, 0.99] | 0; 0.00 [0.00, 0.99] | 0; 0.00 [0.00, 0.99] |
| **Covishield** | 81 (791); 10.24% | 1.07% | 1.86 | 1; 0.25 [0.01, 1.62] | 0; 0.00 [0.00, 0.99] | 0; 0.00 [0.00, 0.99] |
| **Sputnik** | 29  (608); 4.76% | 0.32% | 0.57 | 0; 0.00 [0.00, 0.99] | 0; 0.00 [0.00, 0.99] | 0; 0.00 [0.00, 0.99] |

**Supplementary Table 4: Effects of vaccination in Front-line Workers (FLW) and Health Care Workers (HCW) in Bahrain (Dec 9 to Aug 30).** The comparison to the deployment in the general population was calculated by dividing the rate of vaccination in this population for a given vaccine and rate of vaccination in the general population. Covishield was overrepresented (1.86), and Sputnik underrepresented (0.57). Note the relatively high rate of infection (10.24%) in the Covishield cohort; however, these infections were not clinically consequential, since they led to few hospitalisations, ICU admissions and no deaths.

| **Vaccine Type** | **Total Rate (Hospitalisations)** | | | **Non-Bahraini** | | | **Bahraini** | | |
| --- | --- | --- | --- | --- | --- | --- | --- | --- | --- |
|  | **Rate (Total)** | **LCL UCL** | **OR** | **Rate (Total)** | **LCL UCL** | **OR** | **Rate (Total)** | **LCL UCL** | **OR** |
| **Unvaccinated** | 13.66 (8340) | 13.30 14.02 | NA | 9.65 (2723) | 9.21 10.11 | NA | 17.1 (5617) | 16.57 17.64 | NA |
| **Sinopharm** | 6.94 (1683) | 6.53 7.37 | 2.12 | 3.79 (361.0) | 3.31 4.32 | 2.71 | 8.97 (1322) | 8.38 9.59 | 2.09 |
| **Sputnik V** | 2.24 (77) | 1.66 2.96 | 6.89 | 1.36 (21) | 0.74 2.28 | 7.72 | 2.96 (56) | 2.07 4.08 | 6.77 |
| **Pfizer-BioNtech** | 1.99 (40) | 1.30 2.91 | 7.78 | 1.60 (19) | 0.84 2.73 | 6.58 | 2.57 (21) | 1.41 4.28 | 7.82 |
| **Covishield** | 1.52 (45) | 1.02 2.18 | 10.2 | 1.31 (37) | 0.84 1.95 | 8.02 | 5.76 (8) | 2.08 12.38 | 3.37 |

**Supplementary Table 5, Hospitalisation (%) of SARS-COV-2 PCR positive cases (18+ Ages).** Comparisons of rates of hospitalisation for Bahrainis versus non-Bahraini residents (all registered in the central ISIEH database) including unvaccinated, and 4 vaccines. Note the rates for hospitalisation for non-Bahrainis is lower across all categories. For example, for the Sinopharm vaccine, the rate of hospitalisation was 3.79 percent of all PCR positive cases for non-Bahrainis while it was 8.79 for Bahrainis (effect size= 2.3 fold, p<0.01). All p-values are <0.01 in pairwise comparison between Bahrainis and non-Bahrainis across all the 4 vaccines.
